# The Use of NMR to Study Transient Carbohydrate—Protein Interactions

**DOI:** 10.3389/fmolb.2018.00033

**Published:** 2018-04-11

**Authors:** Pedro M. Nieto

**Affiliations:** Glycosystems Laboratory, Instituto de Investigaciones Científicas, cicCartuja, CSIC/USE, Seville, Spain

**Keywords:** NMR, protein–carbohydrate interaction, STD-NMR, transfer-NOESY, transient interactions

## Abstract

Carbohydrates are biologically ubiquitous and are essential to the existence of all known living organisms. Although they are better known for their role as energy sources (glucose/glycogen or starch) or structural elements (chitin or cellulose), carbohydrates also participate in the recognition events of molecular recognition processes. Such interactions with other biomolecules (nucleic acids, proteins, and lipids) are fundamental to life and disease. This review focuses on the application of NMR methods to understand at the atomic level the mechanisms by which sugar molecules can be recognized by proteins to form complexes, creating new entities with different properties to those of the individual component molecules. These processes have recently gained attention as new techniques have been developed, while at the same time old techniques have been reinvented and adapted to address newer emerging problems.

## Introduction

Glycobiology, which can be defined as the study of the structure, chemistry, biosynthesis, and biological functions of glycans and their derivatives, is fundamental in many critical biological processes (Varki, 1993, 2017). A challenge of structural glycobiology is to reconcile the large variety of 3D shapes that carbohydrates can assume with the high degree of selectivity found between closely related glycans (DeMarco and Woods, 2008). In addition, the binding constants between individual carbohydrates and proteins are generally low. Under these conditions, most of the interactions are in the fast exchange region of the NMR chemical shift scale and a single averaged set of signals is detected. This behavior can be exploited for structural elucidation. The rationale behind transient or transfer experiments is that if the equilibrium is fast enough and the property is dependent on the correlation time, it is possible to observe properties characteristic of the bound state in the averaged ligand signals, due to the larger correlation time of the bound form (Hyde et al., 1980; Feeney, 2000; Meyer and Peters, 2003).

In order to understand the evolution of NMR methods for the study of glycans, the scarcity of simple methods for obtaining isotopically-labeled glycans needs to be kept in mind. When such methods are available, the use of isotope-filtered/edited NMR experiments should be considered. When there is no such alternative, however, transfer techniques need to be employed. These can be applied to systems in fast equilibrium in the relaxation time scale and to properties weighted by the correlation time. Under these circumstances, the average of the NMR property obtained is biased toward the minor population of the bound carbohydrate due to its larger correlation time, even in the presence of an excess of free ligand. This is the basis of the transfer NOE technique and its analogs (Hyde et al., 1980; Ni, 1994). The advent of methodological improvements, monodimensional analogs with better signal to noise ratio, has effectively relaunched the transfer techniques (Stott et al., 1997). Other parameters that can be used to study molecular complexes in a transient state are T_1sel_ (selective longitudinal relaxation time), or T_2_ (transversal relaxation time) and ligand-detected ^1^H relaxation dispersion because they depend on the correlation time, which is a function of the size of the molecule. Theoretical descriptions of T_1_ and T_2_ and of their application to the analysis of binding constants have been reviewed by Stockman and Dalvit (2002) and Peng et al. (Lepre et al., 2004).

A further experiment that benefits from the difference in the correlation times is the STD (saturation transfer difference) experiment (Mayer and Meyer, 1999; Meyer and Peters, 2003). This experiment uses the faster transfer of saturation from the receptor, caused by its long correlation time, that is further transferred to the ligand within the complex and that finally is observed in signals corresponding to the free-state. Since its formulation, the STD technique has experienced a rapid growth, both in its range of applications and in the number of labs using it. Finally, WaterLOGSY uses protonated water molecules to distinguish those that are in fast exchange from those that are buried in the interface with the receptor and therefore reflects the interaction surfaces between receptor and ligand.

## NMR characteristics of free carbohydrates

In general, the principal sources of glycans are *via* chemical synthesis or natural product isolation. In each case, they are almost completely restricted to non-isotopically-labeled compounds. As, from an NMR viewpoint, there are few experimental restrictions for unlabeled-carbohydrates, the analysis conditions must nevertheless be carefully documented and quantified.

### Coupling constants

**Figure 1:**
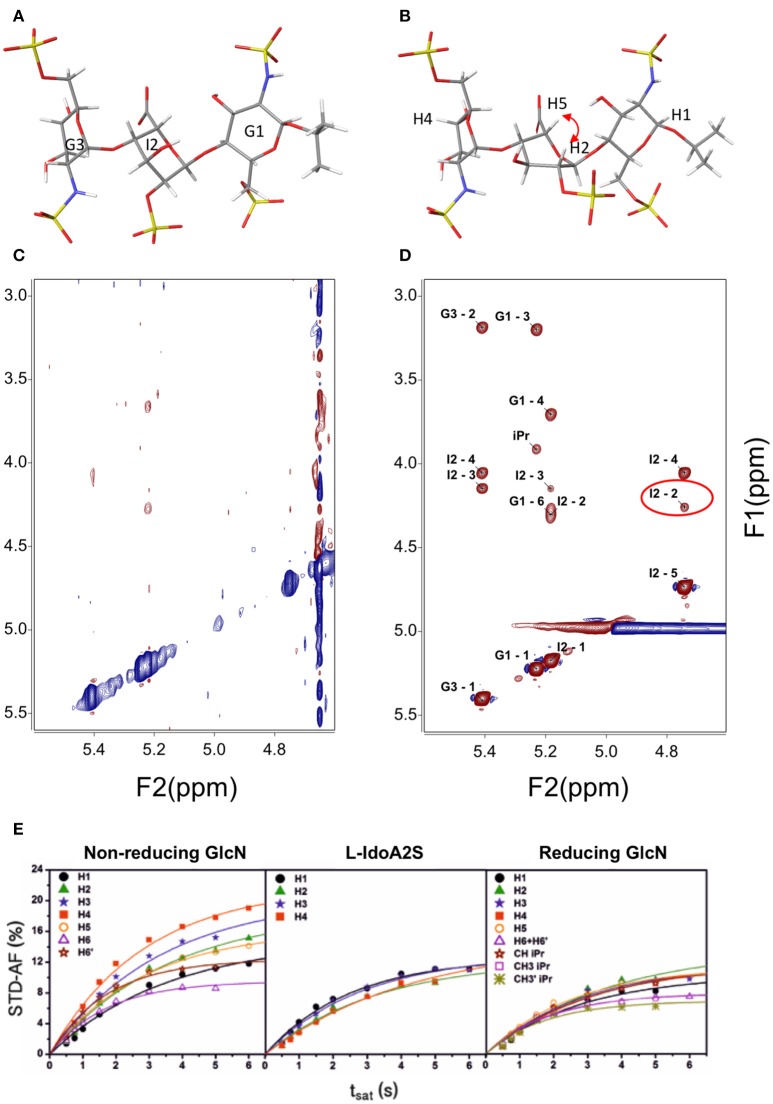
Structure of a heparin trisaccharide with the central iduronate in: **(A)** ^1^C_4_ and **(B)** ^2^S_O_ conformation; **(C)** NOESY 500 MHz and 600 ms t_mix_; **(D)** transfer NOESY (800 MHz, 200 ms t_mix_)—the labels correspond to the assignment of cross-peaks—notice the peak I2 H2—H5, a NOESY peak exclusive to the ^2^S_O_ conformation; and **(E)** STD—affinity factor growing curves for the extraction of the initial rates for quantitative analysis (Muñoz-García et al., 2015).

Three-bond H – H coupling constants depend on the dihedral angles, and consequently they are key to determining the ring conformation. In general, most hexoses are monoconformational and are generally in a ^4^C_1_ or ^1^C_4_ chair conformation. Due to the cyclic nature of the sugars, many redundant interprotonic three-bond coupling constants are available and it is relatively simple to define the conformation from the Cremer–Pople polar coordinates (Cremer and Pople, 1975). Residual dipolar coupling constants provide also data about the relative orientation of two vectors in partially oriented media (Martin-Pastor and Bush, 2001). In carbohydrates, few examples of conformational equilibrium are known; however, one such example is the iduronate residue, which can exist in at least three conformations, ^1^C_4_, ^4^C_1_, and ^2^S_O_, easily distinguishable by their coupling constants. Generally, internal iduronate residues are found in fast equilibrium between ^1^C_4_ and ^2^S_O_ (Figure 1A,B) (Mulloy et al., 1993). This equilibrium is fundamental in the molecular recognition events in which heparin is involved (Canales et al., 2005) and also has been studied by residual dipolar coupling (Jin et al., 2009)

### NOE, distances: the ISPA (isolated spin pair approach)

NOE (and other related parameters such as ROE, transverse NOE, and off-resonance ROE)-based distance constraints are often used to study the glycosidic angle behavior and can thus be used for the determination of the 3D shapes of saccharides. The scarcity of experimental restrictions in the analysis of carbohydrates implies a more accurate quantification of the NOE. Fortunately, for small molecules, distances are easily quantifiable from the NOE by means of an analysis of the growing NOE curve at several mixing times (Macura et al., 1986). Then, assuming equal motional behaviors for an undetermined distance and for a known reference, the initial NOE growth rate (cross-relaxation rate) relationship is inversely proportional to the sixth power of the interprotonic distance (Neuhaus and Williamson, 2000).

Applications of this methodology have increased since the arrival of modern selective 1D NOESY methods based on the double pulsed field gradients spin echo technique (Stott et al., 1997). This scheme has advantages over the bidimensional analogs; it is faster and has better resolution. Moreover, as the protons are relaxing by T_1sel_ instead of T_1_, the growing curves have longer linearity, and thus better-fitting and more precise results can be obtained (Hu and Krishnamurthy, 2006; Munoz-Garcia et al., 2013). The technique has also been applied to off-resonance ROESY, so as to obtain reference-independent distances. Using this approach, it has been possible to obtain the interglycosidic distances for a strongly anisotropic heparin hexasaccharide in which the interprotonic correlation time depends upon the orientation of the vector relative to the axis of the molecule (Munoz-Garcia et al., 2013).

## NMR characteristics of bound small glycans

The ability of NMR to observe and analyze specific signals of individual atoms, focusing analysis on a particular aspect of a complex without the need to solve its entire structure, is the most important consideration.

A bound ligand has NMR properties that are governed by its geometry in the bound state and these can therefore be different to those of the ligand in its free state. In a situation of fast equilibrium in the NMR timescale, the magnitude of the parameter that is measured corresponds to the weighted average of the NMR properties of the free and the bound states. For the special case of correlation time-dependent properties, the weight of the properties derived from the complexed ligand is so large that it is predominant in the averaged values, even at low molar ratio (Ni, 1994; Neuhaus and Williamson, 2000).

### Transfer NOE (TR-NOE)

Most conformational analyses of sugars have been based on NOE data. For the determination of the structures of ligands bound to receptor proteins, transfer NOE (TR-NOE)-based experiments are an excellent tool (Ni, 1994). In suitable conditions, a fast equilibrium in the NMR timescale is established between the free ligand (a small to medium-sized fast-tumbling molecule with a short correlation time) and the ligand bound to the high-molecular-weight receptor (a large and slow-tumbling molecule with a long correlation time) and therefore behaving as the receptor, with a large and fast-growing negative NOE. This averaged situation is reflected in the observation of a large-molecule negative NOE in the signals of the ligand (see Figures 1C,D). In general, modifications of the magnetic field strength or changes in the sample conditions such as temperature or viscosity can be used for fine adjustment if the free ligand is not clearly in the fast-tumbling regime.

In such a case, it is possible to obtain the bound ligand NOE by the analysis of the ligand signals (Ni, 1994; Neuhaus and Williamson, 2000). As a result, the qualitative analysis considering the changes between the free ligand conformation and that of the bound ligand is direct. On the other hand, the quantitative interpretation of the spectrum suffers from the potential effects of spin diffusion. In this situation, complete relaxation matrix calculations (CORCEMA or Mardigras (Borgias and James, 1990; Jayalakshmi and Krishna, 2002) should be used.

In principle, any experiment that measures interprotonic relaxation rates can be used for the analysis of the structure of the bound ligand. Thus, ROESY has been used to improve the experiment, taking advantage of its lower sensitivity to spin diffusion (Poveda et al., 1997). As the ROESY experiment suffers from some spurious effects, transverse-ROESY (Hwang and Shaka, 1992), is better suited as it is less prone to spin-lock artifacts. In addition, the quiet-NOESY experiment has been used. In this experiment, in the middle of the mixing time a biselective signal inversion is applied and a selective band inversion pulse is included, which avoids diffusion through the use of inverted spins (Zwahlen et al., 1994).

Special mention should be made of the monodimensional analogs (Stott et al., 1997). By selecting a single signal, not only is a better resolution achieved due to the change from 2D to 1D but a new dependence on T_1sel_ is created and the NOE exhibits a longer linearity, a feature that is particularly useful when precise distances are required (Hu and Krishnamurthy, 2006).

### STD-NMR

The STD-NMR (Mayer and Meyer, 1999) experiment consists of the difference between two experiments. It is undertaken with low-power irradiation during the relaxation delay on a sample in equilibrium that comprises a large excess of ligand(s) (from 10:1 up to 1,000:1) relative to the receptor (a large molecule), which is present at low concentration (nM to μM). In one experiment, recorded as the reference, dummy irradiation far from the signals is performed, while in the other experiment some signals from the receptor are selectively irradiated. If binding occurs, magnetization from the receptor is transferred to the ligand through close contacts with the receptor in the complex, and the effect will emerge in the difference experiment (Mayer and Meyer, 1999; Meyer and Peters, 2003).

Since its formulation, several applications have been explored for STD-NMR. The first application proposed for the technique was ligand screening; this consists of the deconvolution of a library of potential binding molecules by using several compounds at once in each experiment (Henrichsen et al., 1999). The molecules that bind better will show STD signals. Epitope mapping was the next application proposed. The experiment can be used to determine the relative importance of diverse regions of the ligand in the interaction with the receptor (Mayer and Meyer, 2001). The technique can also be used for affinity constant evaluation. The first application in which calculation of a binding constant was described used the Cheng and Prusoff equation, since the STD values are biased by the relaxation properties of the ligand proton being considered (Meyer and Peters, 2003). Latterly, the initial growth rate of the STD affinity factor has been used, in order to avoid this dependence upon the relaxation of the protons, see Figure 1E (Angulo et al., 2010). One of the potential complications in the STD is peak overlap that can make difficult the interpretation of the results. One of the solutions relies on adding another transfer step, then the peaks will be spread in two dimensions: STD-TOCSY (Mayer and Meyer, 1999) and STD-HMQC (Vogtherr and Peters, 2000). Similarly, spectral editing can also be performed using other nuclei ^15^N or ^19^F (Kövér et al., 2007; Diercks et al., 2009).

**Figure 2:**
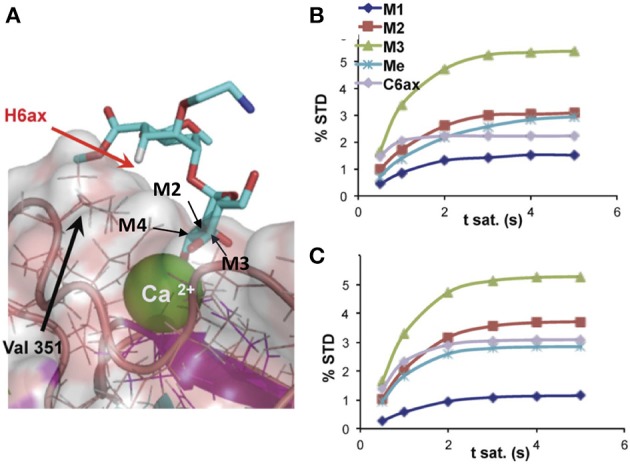
**(A)** Detail of the binding-site structure of a mannose pseudodisaccharide bound to DC-SIGN ECD (ExtraCellular Domain), showing the interactions between the Ca^2+^ ion and the terminal mannose through hydroxyls 3 and 4, and STD growing rates for selected protons; **(B)** experimental; and **(C)** CORCEMA-calculated using the structure shown in **(A)** (Thepaut et al., 2013).

Using methods based on the relaxation matrix, it is possible to calculate the theoretical STD of a given complex. Thus, a quantitative analysis of the ligand within the complex can be carried out using a 3D structure. Based on a 3D model of the complex, using some NMR parameters and considering the saturation time and the irradiation frequency among others, CORCEMA-ST can estimate the different values of saturation transferred from the protein to a particular proton of the ligand (Jayalakshmi and Krishna, 2004, 2005). An example is given in Figure 2. This methodology has also been extended to the case of multiple binding modes within the same binding site (Angulo et al., 2008) and to the iterative refinement of a complex structure (Jayalakshmi and Krishna, 2005).

### Selective T_1_ (T_1sel_) and T_2_

T_1sel_ and T_2_ also depend on correlation times and in principle they can be used to study association processes for fast equilibrium between ligand and receptor, observing the ligand signals at low concentration of the receptor. These values can be used for the analysis of binding constants by performing a titration (García-Jiménez et al., 2017). A series of T_1sel_ /T_2_ values are obtained at different ratios of ligand to receptor, and by fitting these to the variation in the ligand/receptor ratio, K_D_ or IC_50_ can be extracted. Recently the application of ^1^H relaxation dispersion measurements has been described for weak binding systems using both T_2_ and T_1ρ_ (Moschen et al., 2015; Trigo-Mouriño et al., 2017).

### WaterLOGSY

The WaterLOGSY experiment (Dalvit et al., 2000) takes into account explicitly the water molecules surrounding the macromolecular complex. It is easy to implement and consists essentially of a selective excitation of the water molecules in a sample prepared in H_2_O, followed by a NOESY mixing period and a water suppression scheme for detection (Dalvit et al., 2001).

This experiment traces the mechanisms that follow the magnetization from excited water to ligand protons. It can be a direct transfer of magnetization from bulk water to ligand, through exchangeable protons from the receptor or via water molecules buried into the receptor interface. These molecules have tumbling times closer to the receptor than to the bulk water or ligand. Due to the slow correlation times of the bound ligand or the buried water molecules the peaks through the two later mechanisms will have the opposite sign as the direct transfer of magnetization of the free compounds (Stockman and Dalvit, 2002).

The WaterLOGSY competition experiment is used for ligand library deconvolution, in which several relay pathways are used constructively, transferring bulk water magnetization to the ligand in a selective manner (Dalvit et al., 2002). In this experiment, the resonances of non-binding compounds appear with opposite sign, corresponding to NOE in the narrowing limit, and they tend to be weaker than those of the interacting ligands.

## New developments

A recent application of the WaterLOGSY is the LOGSY titration (Geist et al., 2017). From the slopes of the WaterLOGSY vs the protein concentration curves, LOGSY-titration factors can be obtained, that can be used for epitope mapping alternative to STD-AF_0_. In this case, a great advantage is that isolated water molecules are always in the narrowing limit of the NOE curve.

Complementary information can be also obtained using paramagnetic tags attached to carbohydrates. At least two methods can be exploited: PCS, where changes in chemical shifts are produced and PRE, where additional relaxation is caused. In both cases the effects are proportional to the distance to the paramagnetic center. The paramagnetic center can be either a Lanthanide (Gao et al., 2016; Canales et al., 2017) or nitroxide carrying spin labels like TEMPO.

## Integrating the results. from NMR data to 3D structures

The process of deriving a 3D structure from NMR data is not straightforward and it requires the application of force field modeling, with experimental constraints taken from the experimental data. For carbohydrates, accurate distances have to be used and complemented by high-quality theoretical data; this is because an accurate description of the flexibility is required. This often implies the use of explicit solvent MD (molecular dynamics) calculations for the free ligand.

Several force field programs have been developed for carbohydrates, the most widely used being Amber, making use of the parameters for carbohydrates developed by Woods (GLYCAM) (Kirschner et al., 2008); CHARMM (Brooks et al., 2009), optimized by Mackerel; and GROMACS (Hess et al., 2008). These force fields are compatible with other force fields developed for proteins and peptides and can be used in mixed systems calculations.

Using the experimental NMR data from a complex, we could obtain a 3D structure of the ligand within the complex using transfer NOESY evaluation, or at least qualitative data relative to the potential conformational changes upon binding (Ni, 1994). Finally, CORCEMA-ST software is capable of calculating the STD values for individual protons (Krishna and Jayalakshmi, 2008), and of verifying the bound ligand conformation. STD_0_-AF have been used for the definition of the structure, position, and conformation of the ligand within the complex (Enríquez-Navas et al., 2011). This approach can also be used to tackle more complex situations, such as multiple binding modes in one or more than one site (Angulo et al., 2008). Finally, it has been used to refine NMR structures, using STD-NMR intensity-restrained optimization (Jayalakshmi and Krishna, 2005).

## Future perspectives

NMR provides tools for the analysis and study of low-resolution 3D structures of protein–carbohydrate complexes. A particular advantage is that there are no upper limits to the size of the receptor and the analyses can be performed even with complete cells. In order to improve the reliability of the results, however, efforts should be made to achieve an integrated semi-quantitative method combining all the transfer NMR techniques that can deliver spatial resolution, namely transfer NOESY, STD-NMR, and WaterLOGSY. Recently, promising new methods based on PCS (Pseudo Contact Shifts) have emerged. In the case of the lanthanide paramagnetic tags using PCS they can be applied to protein–carbohydrate complexes with the advantage of the larger dispersion of the signals induced by the lanthanide linked to the carbohydrate (Canales et al., 2017) or the protein (Gao et al., 2016).

## Author contributions

The author confirms being the sole contributor of this work and approved it for publication.

### Conflict of interest statement

The author declares that the research was conducted in the absence of any commercial or financial relationships that could be construed as a potential conflict of interest.
